# Urban-Rural Differences in the Association of eHealth Literacy With Medication Adherence Among Older People With Frailty and Prefrailty: Cross-Sectional Study

**DOI:** 10.2196/54467

**Published:** 2024-09-11

**Authors:** Ying Guo, Zixuan Hong, Chenglin Cao, Wenwen Cao, Ren Chen, Jing Yan, Zhi Hu, Zhongliang Bai

**Affiliations:** 1 Department of Health Services Management, School of Health Services Management, Anhui Medical University, Hefei, China; 2 Key Laboratory of Public Health Social Governance, Philosophy and Social Sciences of Anhui Province, Hefei, China

**Keywords:** eHealth literacy, medication adherence, frailty, older people, China

## Abstract

**Background:**

With advances in science and technology and improvements in health literacy, more studies have focused on frailty prevention by promoting medication adherence, emphasizing the role of eHealth literacy. However, the association between eHealth literacy and medication adherence in frail older adults has not been well studied, and it is unknown whether urban-rural differences exist in this relationship.

**Objective:**

This study aims to examine the relationship between eHealth literacy and medication adherence in older people with different frailty statuses, emphasizing variations between rural and urban areas.

**Methods:**

Between November and December 2020, a total of 4218 urban and rural community members (aged ≥60 years) in China were recruited as participants using a multistage random sampling method. A face-to-face structured questionnaire survey was conducted to collect information on demographic characteristics, eHealth literacy (consisting of application, evaluation, and decision dimensions), and medication adherence. eHealth literacy was assessed using the Chinese version of the eHealth Literacy Scale developed by Norman and Skinner, and medication adherence was measured using the 4-item Morisky scale. We used a general descriptive analysis and stratified logistic regression models to examine how eHealth literacy is linked to medication adherence and urban-rural differences.

**Results:**

There were 4218 respondents, of which 2316 (54.9%) lived in urban areas and 1902 (45.1%) in rural areas, respectively. After adjusting for potential confounders, among participants with prefrailty, eHealth literacy was associated with medication adherence in urban areas in terms of less application (adjusted odds ratio [AOR] 1.16, 95% CI 0.82‐1.63), less evaluation (AOR 1.29, 95% CI 0.92‐1.81), and less decision ability (AOR 1.20, 95% CI 0.86‐1.68); eHealth literacy was linked with medication adherence in the rural areas in terms of less application (AOR 1.10, 95% CI 0.56‐2.13), less evaluation (AOR 1.05, 95% CI 0.61‐1.79), and less decision ability (AOR 1.10, 95% CI 0.64‐1.90). Among frail participants, less eHealth literacy (AOR 0.85, 95% CI 0.48‐1.51), along with its dimensions, including less application (AOR 0.85, 95% CI 0.47‐1.54), evaluation (AOR 0.89, 95% CI 0.50‐1.57), and decision ability (AOR 0.99, 95% CI 0.55‐1.76), were associated with medication adherence in urban areas; less eHealth literacy (AOR 0.89, 95% CI 0.48‐1.65), along with its dimensions, including less application (AOR 1.23, 95% CI 0.62‐2.44), evaluation (AOR 0.98, 95% CI 0.53‐1.82), and decision ability (AOR 0.90, 95% CI 0.49‐1.67), were associated with medication adherence in rural areas.

**Conclusions:**

The results of this study suggest that there is an association between eHealth literacy and medication adherence among older people with frailty and prefrailty. To promote medication adherence, eHealth literacy can be helpful in tailoring interventions.

## Introduction

Population aging has become a common phenomenon worldwide and is increasing in East Asian countries. For example, the aging rate in Japan is expected to exceed 30% by 2030 [1]. China, one of the fastest aging countries in the world, is predicted to have over 402 million people aged ≥60 years by 2040. Under such background, frailty, an age-related geriatric syndrome, has become a global public health concern [2,3]. Frailty is a clinical condition characterized by an individual’s excessive vulnerability to stress, increasing the risk of adverse health outcomes (eg, surgical complications, disability, and fatality) in older adults [4] and reducing their quality of life [5,6].

Furthermore, previous research revealed that polypharmacy and irrational medication behaviors significantly increase the risk of frailty [7]. Additionally, as the vast majority of frail patients have multiple comorbidities and require long-term medication, the medical and economic burden on the family and society is increased [2]. Therefore, proper and effective measures to improve medication adherence among frail communities have become an urgent issue [8,9].

Medication adherence refers to the patient’s compliance to take medications as prescribed and directed until cured or until the condition has improved sufficiently [10]. Research has demonstrated poor medication adherence among older Chinese people, which leads to a decline in physical functioning and reduces their quality of life [11-13]. Additionally, a study found that frequent dissemination of health information on the internet is beneficial for cultivating good drug habits among patients (eg, taking medication on time and taking appropriate medication) [14]. Therefore, the role of eHealth literacy in improving medication adherence should be given more attention.

eHealth literacy is a multifaceted concept that describes the knowledge reserve of individuals to retrieve, understand, and evaluate health information in electronic resources as well as the ability to use this information to solve health problems [15]. Literacy comprises the application, judgment, and decision-making abilities to use health information and services [16]. In recent decades, there has been a growing focus on the positive impact of eHealth literacy on promoting healthy behaviors among patients with various diseases [17], especially medication adherence for older adults challenged by chronic diseases [18]. Additionally, studies have disclosed that in patients with hypertension and heart disease, a higher level of eHealth literacy is associated with greater drug knowledge and better their compliance with physicians’ orders [18,19]. Nevertheless, little research has been conducted on the relationship between eHealth literacy and medication adherence in older people with frailty.

With the growing urbanization, disparities in socioeconomic development between urban and rural areas have become increasingly apparent, resulting in more severe health inequities [20]. Previous research has found that older adults in urban areas showed better self-rated health overall compared to those in rural areas, due to a higher frequency of exercise and more significant social activity among urban counterparts [21]. Furthermore, urban-rural differences are reflected in eHealth literacy. For instance, a study showed that older rural people had lower literacy in eHealth due to their economic and educational levels, which hampered them from using, searching for, and identifying the correct health information in electronic resources [22]. Moreover, there are differences in medication adherence between urban and rural older adult populations. Compared with older people in urban areas, adherence to osteoporosis medications was relatively low among those in rural areas. On the contrary, older people in rural areas showed better compliance with antihypertensive drugs compared to those in urban areas [23]. Consequently, it is necessary to investigate the relationship between eHealth literacy and medication adherence in older adults with frailty or prefrailty and to explore the disparities between urban and rural settings.

Currently, the association between eHealth literacy and medication adherence in older adults with frailty or prefrailty has not been well examined. In light of this, this study aimed to explore the relationship between eHealth literacy and medication adherence in older people with different frailty statuses, emphasizing variations between rural and urban areas. The evidence from this study may further contribute to developing personalized measures to improve medication adherence among older people by improving eHealth literacy, which is critical for reducing rural-urban health inequalities and promoting healthy aging.

## Methods

### Participants

To collect a representative sample of eligible participants, a multistage stratified random sampling approach was used between November and December 2020. The Yangtze River Delta region of China’s Shanghai, Zhejiang Province, Jiangsu Province, and Anhui Province were the sampling regions during the initial stage. Next, a simple random sampling method was used to select 2 county-level regions in each sample area. In the second stage, we randomly selected 1 or 2 townships and urban streets, and 16 street communities were included in the investigation. At last, in every chosen street and township community, 24 neighborhood committees and villages were selected as sample locations.

In this study, according to the household registration and residence information, participants were categorized as part of the urban population if they were registered or resided in the city, while the remaining ineligible participants identified as the rural population. Given the focus of this study on the frail older population and the difference between urban and rural areas, we included the older population aged 60 years and older who had lived in the locality for more than 3 years. Older adults who could not communicate with the researchers or had cognitive impairments were excluded. Among the 4257 older adults, 4218 were included in this study. Our previous published papers have provided more detailed information on the study design, data collection, and participant recruitment [11,24,25].

### Ethical Considerations

Prior to the start of the study, the purpose and procedure of the interview were explained to the participants, and all participants signed informed consent forms. The Biomedical Ethics Committee of Anhui Medical University has approved and filed the research content, investigation methods, research methodology, and informed consent involved in this study (No. 20150297).

### Measurement of Frailty Status

To evaluate the frailty status of respondents, we used a questionnaire comprising 4 dimensions and 23 items. We summed the 23 items to get the total frailty status score before dividing the total frailty score by 23 to obtain the frailty status score. According to previous studies [11,26], frailty was divided into 3 groups: nonfrailty (<0.12), prefrailty (0.12‐0.24), and frailty (≥0.25) based on frailty scores. Good internal consistency was demonstrated by Cronbach *α* of 0.771 in this sample. Additionally, the specific measurement details of this tool can be viewed in the attachment (Multimedia Appendix 1).

### Measurement of Medication Adherence

In accordance with prior studies [10,27], the 4-item Morisky scale [28-30] was used to measure medication adherence, the main dependent variable. The results included the following two aspects: if participants answered “No” to all questions in the questionnaire, they were classified as having good medication adherence; otherwise, they were classified as having poor medication adherence. The validity of this scale was also previously reported [11]. For details on this measurement tool, please refer to the supplemental file here (Multimedia Appendix 1 [28-30]).

### Measurement of eHealth Literacy

This study adopted the eHealth Literacy Scale (eHEALS) created by Norman and Skinner [31], which has been translated into Chinese and widely used to assess eHealth literacy among Chinese older adults and has demonstrated excellent reliability and validity (Cronbach *α*=0.992) [16,32,33]. The eHEALS consists of 3 dimensions and 8 items, namely, web-based health information and services application ability (items 1‐5), judgment ability (items 6‐7), and decision-making ability (item 8). A 5-point Likert scale was used (ranging from 1=“very inconsistent” to 5=“very consistent”); the total score ranged from 8 to 40. The total score of the 8 items was calculated as the eHealth literacy score, with higher scores indicating higher levels of eHealth literacy. Please refer to the supplementary file for details on this measurement tool (Multimedia Appendix 1).

### Measurement of Related Variables

During data collection, we obtained information on demographic and health-related variables. These variables included age, gender, BMI, residence, educational attainment, living arrangement, and marital status. Additionally, information was collected regarding sources of income, smoking and drinking habits, depression status, and the level of functional ability (robust or limited) of the participants.

### Statistical Analysis

In the first step, we used simple descriptive analyses to describe the characteristics of the sample. General characteristics were expressed as a percentage of categorical variables. In the second step, we developed stratified logistic regression models to explore urban-rural differences in the correlation between eHealth literacy and medication adherence. Next, after adjusting for potential covariates following the literature [11,18,34], the odds ratio, adjusted odds ratio (AOR), and 95% CI were used to present the results of these models.

In this study, SPSS 23.0 (IBM Corp) was used for data analysis. *P*<.05 was used for statistical significance threshold.

## Results

### Descriptive Statistics Results

Table 1 displays the participant characteristics separated by place of residence: urban (2316/4218, 54.9%) and rural (1902/4218, 45.1%). Of these participants, the 60‐69–year age group accounted for 42.2% (1779/4218) of the participants; 64.8% (2734/4218) were women; 78.8% (3325/4218) were married or cohabiting; 86.5% (3650/4218) lived with others; over half (2571/4218, 61.0%) of the participants had education levels at or below primary school. Regarding health behavior, most participants reported never smoking (3332/4218, 79.0%) or never drinking (3386/4218, 80.3%). Regarding health status, 55.8% (2355/4218) of the participants were not depressed, 45.6% (1923/4218) were functionally limited, and more than half (2319/4218, 55.0%) were in a frail or prefrail stage. Most participants were at lower levels of application, evaluation, and decision dimensions. In terms of total eHealth literacy score, 18.9% (797/4218) were at a high level of eHealth literacy, while 81.1% (3421/4218) were at a low level of eHealth literacy.

**Table 1.** Characteristics of the participants aged ≥60 years by residence (N=4218).

Characteristics		Residence		
		Urban (n=2316), n (%)	Rural (n=1902), n (%)	Total (n=4218), n (%)
**Age (years)**				
	60‐69	997 (43.0)	782 (41.1)	1779 (42.2)
	70‐79	880 (38.0)	858 (45.1)	1738 (41.2)
	≥80	439 (19.0)	262 (13.8)	701 (16.6)
**Gender**				
	Male	861 (37.2)	623 (32.8)	1484 (35.2)
	Female	1455 (62.8)	1279 (67.2)	2734 (64.8)
**BMI (kg/m** ^**2**^ **)**				
	≤18.5	103 (4.4)	107 (5.6)	210 (5.0)
	18.5‐22.9	709 (30.6)	674 (35.4)	1383 (32.8)
	23‐27.4	1184 (50.9)	875 (46.0)	2059 (48.8)
	≥27.5	320 (13.8)	246 (12.9)	566 (13.4)
**Living status**				
	Living with others	1983 (85.6)	1667 (87.6)	3650 (86.5)
	Living alone	333 (14.4)	235 (12.4)	568 (13.5)
**Marital status**				
	Married/cohabited	1827 (78.9)	1498 (78.8)	3325 (78.8)
	Single	489 (21.1)	404 (21.2)	893 (21.2)
**Education level**				
	Primary school and below	980 (42.3)	1591 (83.6)	2571 (61.0)
	Junior school	708 (30.6)	229 (12.0)	937 (22.2)
	High school and above	628 (27.1)	82 (4.3)	710 (16.8)
**Smoking status**				
	Former smoker	199 (8.6)	108 (5.7)	307 (7.3)
	Smoker	312 (13.5)	267 (14.0)	579 (13.7)
	Nonsmoker	1805 (77.9)	1527 (80.3)	3332 (79.0)
**Drinking status**				
	Former drinker	116 (5.0)	74 (3.9)	190 (4.5)
	Drinker	361 (15.6)	281 (14.8)	642 (15.2)
	Nondrinker	1839 (79.4)	1547 (81.3)	3386 (80.3)
**Income**				
	Salary	67 (2.9)	323 (17.0)	390 (9.2)
	Pension	2048 (88.4)	296 (15.6)	2344 (55.6)
	Family providing	66 (2.8)	778 (40.9)	844 (20.0)
	Subsidy	108 (4.7)	352 (18.5)	460 (10.9)
	Others	27 (1.2)	153 (8.0)	180 (4.3)
**Depressive status**				
	No depression	1275 (55.1)	1080 (56.8)	2355 (55.8)
	Minimal to mild depression	1011 (43.7)	793 (41.7)	1804 (42.8)
	Depression	30 (1.3)	29 (1.5)	59 (1.4)
**Endowment insurance**				
	None	119 (5.1)	419 (22.0)	538 (12.8)
	Basic endowment insurance for the urban working group	1788 (77.2)	159 (8.4)	1947 (46.2)
	Pension insurance for flexible employees	10 (0.4)	3 (0.2)	13 (0.3)
	Social endowment insurance for nonworking urban residents	378 (16.3)	213 (11.2)	591 (14.0)
	New rural social endowment insurance for rural residents	15 (0.6)	1095 (57.6)	1110 (26.3)
	Commercial endowment insurance	6 (0.3)	13 (0.7)	19 (0.5)
**Functional ability**				
	Robust	1527 (65.9)	768 (40.4)	2295 (54.4)
	Limited	789 (34.1)	1134 (59.6)	1923 (45.6)
**Frailty status**				
	Robust	1147 (49.5)	752 (39.5)	1899 (45.0)
	Prefrail	809 (34.9)	718 (37.7)	1527 (36.2)
	Frail	360 (15.5)	432 (22.7)	792 (18.8)
**Medication adherence**				
	Adequate adherence	1572 (67.9)	1243 (65.4)	2815 (66.7)
	Inadequate adherence	744 (32.1)	659 (34.6)	1403 (33.3)
**Application dimension**				
	High	606 (26.2)	133 (7.0)	739 (17.5)
	Low	1710 (73.8)	1769 (93.0)	3479 (82.5)
**Evaluation dimension**				
	High	626 (27.0)	182 (9.6)	808 (19.2)
	Low	1690 (73.0)	1720 (90.4)	3410 (80.8)
**Decision dimension**				
	High	621 (26.8)	183 (9.6)	804 (19.1)
	Low	1695 (73.2)	1719 (90.4)	3414 (80.9)
**eHealth literacy**				
	High	620 (26.8)	177 (9.3)	797 (18.9)
	Low	1696 (73.2)	1725 (90.7)	3421 (81.1)

### Logistic Regression Models: Relationship Between eHealth Literacy and Medication Adherence for Nonfrail Participants

Table 2 presents the results of logistic regression models after the variables were adjusted. Among urban-dwelling nonfrail participants, eHealth literacy and all its dimensions were observed to be statistically correlated with medication adherence, indicating that the AOR of having poor medication adherence was shown to be 1.50 times (95% CI 1.05‐2.14), 1.47 times (95% CI 1.04‐2.10), and 1.48 times (95% CI 1.03‐2.11) more likely for people with a lower eHealth literacy in terms of application dimension, evaluation dimension, and decision dimension, respectively. However, in rural nonfrail participants, eHealth literacy and its dimensions were not statistically associated with medication adherence.

**Table 2.** Results of binary logistic regression of the association between eHealth literacy and medication adherence in nonfrail participants by residence type (n=1899).

eHealth literacy	Urban						Rural					
	Unadjusted			Adjusted^a^			Unadjusted			Adjusted^a^		
	*B* (SE)^b^	OR^c^	95% CI	*B* (SE)	AOR^d^	95% CI	*B* (SE)	OR	95% CI	*Β* (SE)	AOR	95% CI
Lower application dimension(reference: higher application dimension)	0.23 (0.17)	1.26	0.91‐1.74	0.40 (0.18)	1.50^e^	1.05‐2.14	0.35 (0.38)	1.42	0.68‐2.97	0.40 (0.38)	1.49	0.70‐3.14
Lower evaluation dimension(reference: higher evaluation dimension)	0.22 (0.16)	1.25	0.91‐1.73	0.39 (0.18)	1.47^e^	1.04‐2.10	0.23 (0.32)	1.25	0.67‐2.35	0.25 (0.32)	1.29	0.68‐2.43
Lower decision dimension(reference: higher decision dimension)	0.22 (0.17)	1.25	0.90‐1.73	0.39 (0.18)	1.48^e^	1.03‐2.11	0.26 (0.32)	1.30	0.70‐2.43	0.29 (0.32)	1.34	0.71‐2.53
Lower eHealth literacy score(reference: higher eHealth literacy score)	0.26 (0.17)	1.30	0.94‐1.80	0.44 (0.18)	1.55^e^	1.08‐2.11	0.21 (0.32)	1.23	0.66‐2.30	0.24 (0.32)	1.27	0.68‐2.41

a Adjusted by age, gender, and education.

b *B* (SE): coefficient (standard error).

c OR: odds ratio.

d AOR: adjusted odds ratio.

e *P<*.05.

### Logistic Regression Models: Relationship Between eHealth Literacy and Medication Adherence for Participants With Prefrailty

As Table 3 shows, eHealth literacy (AOR 1.30, 95% CI 0.93‐1.82), in terms of less application (AOR 1.16, 95% CI 0.82‐1.63), less evaluation (AOR 1.29, 95% CI 0.92‐1.81), and less decision ability (AOR 1.20, 95% CI 0.86‐1.68), was associated with medication adherence in urban-dwelling participants with prefrailty. eHealth literacy (AOR 1.01, 95% CI 0.58‐1.76), in terms of less application (AOR 1.10, 95% CI 0.56‐2.13), less evaluation (AOR 1.05, 95% CI 0.61‐1.79) and less decision ability (AOR 1.10, 95% CI 0.64‐1.90), was associated with medication adherence in rural residents with prefrailty.

**Table 3.** Results of binary logistic regression of the association between eHealth literacy and medication adherence in prefrail participants by residence type (n=1527).

eHealth literacy	Urban						Rural					
	Unadjusted			Adjusted^a^			Unadjusted			Adjusted^a^		
	*B* (SE)^b^	OR^c^	95% CI	*B* (SE)	AOR^d^	95% CI	*B* (SE)	OR	95% CI	*B* (SE)	AOR	95% CI
Lower application dimension(reference: higher application dimension)	−0.01 (0.16)	0.99	0.72‐1.35	0.15 (0.17)	1.16	0.82‐1.63	0.06 (0.34)	1.06	0.55‐2.05	0.09 (0.34)	1.10	0.56‐2.13
Lower evaluation dimension(reference: higher evaluation dimension)	0.09 (0.16)	1.10	0.80‐1.50	0.26 (0.17)	1.29	0.92‐1.81	0.01 (0.27)	1.01	0.59‐1.72	0.05 (0.28)	1.05	0.61‐1.79
Lower decision dimension(reference: higher decision dimension)	0.02 (0.16)	1.02	0.75‐1.40	0.18 (0.17)	1.20	0.86‐1.68	0.05 (0.28)	1.05	0.61‐1.81	0.10 (0.28)	1.10	0.64‐1.90
Lower eHealth literacy score(reference: higher eHealth literacy score)	0.10 (0.16)	1.11	0.81‐1.52	0.26 (0.17)	1.30	0.93‐1.82	−0.04 (0.28)	0.96	0.56‐1.67	0.01 (0.28)	1.01	0.58‐1.76

a Adjusted by age, gender, and education.

b *B* (SE): coefficient (standard error).

c OR: odds ratio.

d AOR: adjusted odds ratio.

### Logistic Regression Models: Relationship Between eHealth Literacy and Medication Adherence for Participants With Frailty

Table 4 shows that after adjustment for covariates, 3 dimensions of eHealth literacy were observed to be associated with medication adherence among the urban frail population, indicating that the AOR of experiencing poor medication adherence was 0.85 times (95% CI 0.47‐1.54), 0.89 times (95% CI 0.50‐1.57), and 0.99 times (95% CI 0.55‐1.76) more likely for people with lower eHealth literacy in the application, evaluation, and decision dimensions, respectively. Among rural frail participants, the application dimension (AOR 1.23, 95% CI 0.62‐2.44) was positively correlated with medication adherence, and the eHealth literacy (AOR 0.89, 95% CI 0.48‐1.65), evaluation (AOR 0.98, 95% CI 0.53‐1.82), and decision (AOR 0.90, 95% CI 0.49‐1.67) dimensions were negatively correlated with medication adherence.

**Table 4.** Results of binary logistic regression of the association between eHealth literacy and medication adherence in frail participants by residence type (n=792).

eHealth literacy	Urban						Rural					
	Unadjusted			Adjusted^a^			Unadjusted			Adjusted^a^		
	*B* (SE)^b^	OR^c^	95% CI	*B* (SE)	AOR^d^	95% CI	*B* (SE)	OR	95% CI	*B* (SE)	AOR	95% CI
Lower application dimension(reference: higher application dimension)	−0.19 (0.28)	0.83	0.48‐1.43	−0.16 (0.30)	0.85	0.47‐1.54	0.12 (0.34)	1.13	0.57‐2.21	0.21 (0.35)	1.23	0.62‐2.44
Lower evaluation dimension(reference: higher evaluation dimension)	−0.14 (0.27)	0.87	0.51‐1.47	−0.12 (0.29)	0.89	0.50‐1.57	−0.08 (0.31)	0.92	0.50‐1.69	−0.02 (0.31)	0.98	0.53‐1.82
Lower decision dimension(reference: higher decision dimension)	−0.03 (0.27)	0.97	0.57‐1.65	−0.01 (0.30)	0.99	0.55‐1.76	−0.18 (0.31)	0.84	0.46‐1.54	−0.10 (0.31)	0.90	0.49‐1.67
Lower eHealth literacy score(reference: higher eHealth literacy score)	−0.18 (0.27)	0.84	0.49‐1.42	−0.16 (0.29)	0.85	0.48‐1.51	−0.18 (0.31)	0.84	0.46‐1.54	−0.11 (0.31)	0.89	0.48‐1.65

a Adjusted by age, gender, and education.

b *B* (SE): coefficient (standard error).

c OR: odds ratio.

d AOR: adjusted odds ratio.

## Discussion

### Principal Findings

This study, as far as we know, is the first to explore the association between eHealth literacy and medication adherence and examine the urban-rural differences in this association among older people with frailty and prefrailty. An association was found between eHealth literacy and medication adherence in prefrail and frail older adult populations, but no urban-rural differences existed.

eHealth literacy was associated with medication adherence in the nonfrail older population. All dimensions of eHealth literacy, including application, evaluation, and decision, correlated with medication adherence in nonfrail older people and were statistically significant in urban areas. In other words, higher levels of eHealth literacy lead to better medication adherence. This result may align with previous studies that demonstrated a positive correlation between education levels and eHealth literacy [35,36]. Urban-dwelling older people are more likely to have access to educational opportunities, which contributes to a higher awareness and understanding of health knowledge, further enhancing their ability to access and use digital products. Effective use of electronic devices and acquiring high-quality health information may contribute to understanding drug dosage and use, allowing older adults to make informed decisions [37,38]. Besides, older urban populations are mostly from families of privileged economic status. Previous research has shown that better family financial situations are associated with higher self-perceived health literacy among residents [39]. In short, residents with better family conditions could use more electronic products and have a strong sense of health care and a proactive willingness to use network health care resources.

eHealth literacy and medication adherence were positively correlated, suggesting that more eHealth literacy is more likely to result in better medication adherence in the prefrail older population. This is consistent with previous research that states that high levels of eHealth literacy are a protective factor in promoting medication adherence [18]. Unlike prior studies, our study focused specifically on the vulnerable group of older adults with frailty. Older adults with prefrailty tend to have an increased need to access health services due to physical and psychological problems [40]. At the same time, appointments may be booked and registered through mobile devices, such as mobile phones, which facilitates a range of access behaviors, increasing the use of medical devices and the frequency and ability to find health information via the internet. This encourages the older population to access digital information and improve their eHealth literacy. For example, older people can search for the precautions, dosage, and course of medication on the internet, improving medication adherence.

According to our results, eHealth literacy, including evaluation and decision skills, was negatively correlated with medication adherence in frail older adults, implying that lower levels of eHealth literacy were associated with better medication adherence compared to high levels of eHealth literacy. The following explanations could account for this result. There is a substantial amount of research reporting a heavy physical and psychological burden, including loss of audiovisual function, reduced fine motor control, cognition impairment, dementia, and even death, among older people with frailty [4,41]. These adverse health outcomes may render frail older adults incapable of using electronic devices, reducing their ability to access health information via the internet [42,43]. Simultaneously, older adults with frailty need family companionship and medication monitoring and may receive more attention and help from social networks, such as family and carers. While the time-dependent burden on carers may be higher for more older people with frailty, the involvement of a carer leads to more consistent medication-taking behaviors, objectively reducing the probability of missing or incorrectly taking medication, and thus improving medication adherence.

However, it is concerning that this study did not find urban-rural differences in the association between eHealth literacy and medication adherence in older adults with prefrailty and frailty. There are both macrosocial and microindividual reasons for this outcome. At the macro level, on the one hand, along with the finishing of the building of a moderately prosperous society in all respects and the implementation of poverty alleviation, the most basic production and living needs of the people living in villages have been met, and the infrastructure in impoverished areas have been improved [44,45]. On this basis, China has carried out top-level design and macroplanning for the construction of digital villages, accelerated the bridging of the “digital divide” between urban and rural areas, and given full play to the role of information technology as a driving force in rural revitalization [46]. At this stage, the construction of China’s digital countryside has begun to show results, with existing administrative villages across the country fully realizing the “village to village broadband.” The number of internet users in rural areas is increasing, and the gap between urban and rural areas in terms of access to the internet continues to narrow [47]. Well-established telecommunication networks and infrastructures may provide the foundation for older rural populations to use electronic devices, leading to an increased ability to use digital products and greater confidence in searching for digital health information.

On the other hand, with the deepening of China’s health care system reform and the continuous promotion of the hierarchical medical system, digital health care forms, such as remote consultation, remote treatment, and medical information sharing platforms, will help medical resources eliminate spatial constraints [48]. This helps narrow the gap between urban and rural medical resources, promotes the accessibility of health services, improves the allocation of health resources, and ensures equal use of health care [49,50]. At the same time, promoting telemedicine knowledge and health education activities for older adults in rural areas is increasing trust in telemedicine and improving eHealth literacy among this population.

At the micro level, there is a growing awareness of health care among the older population. Accompanied by the rise of short videos on third-party platforms, such as TikTok, the visual presentation effectively alleviates the dilemma of low literacy rates and difficulty accessing health information among the rural older population [51]. This provides older people with a wide range of health care resources, facilitates access to health information, and improves eHealth literacy and medication adherence.

### Limitations

This study faced several limitations. First, the causal relationships between eHealth literacy and medication adherence might not be appropriately confirmed using this cross-sectional study. Therefore, longitudinal or cohort research is required to validate the current investigation’s results. Second, the survey data came from self-reporting, which was prone to a risk of recall bias due to false or inaccurate responses from participants. Despite these limitations, the advantages of our study include a high response rate, a sizable representative sample size, as well as reliable and valid measurement instruments for data collection. The outcomes of this paper are provocative for developing effective measures to prevent and control the development of frailty among the older population in the future.

### Conclusions

This study reports urban-rural differences in the association between eHealth literacy and medication adherence in prefrail and frail older populations. Our study found an association between eHealth literacy and medication adherence in the prefrail and frail older adult population but no urban-rural differences were found. Although our research was not statistically significant, it is an accurate picture of the urban-rural differences in the association of eHealth literacy and medication adherence in China’s frail older adult population, with rigorous data investigation and statistical analysis, and it can still provide a reference for subsequent related studies. The results of this study need to be further justified by in-depth research, and they may contribute to the development of targeted approaches to improve medication adherence among older adults from an eHealth literacy perspective.

## Supplementary material

10.2196/54467 Multimedia Appendix 1 Questionnaires used in the study.

## Abbreviations

AOR: adjusted odds ratio
eHEALS: eHealth Literacy Scale
