# Supplementary material for: Urban-Rural Differences in the Association of eHealth Literacy With Medication Adherence Among Older People With Frailty and Prefrailty: Cross-Sectional Study
Source: JMIR Public Health Surveill. 2024 Sep 11;10:e54467. doi: 10.2196/54467 (PMC11409828; doi:10.2196/54467)
Supplement: Multimedia Appendix 1 [file publichealth-v10-e54467-s001.docx]

**Frailty assessment Scale**

**1. Body Frailty**

**1.1** Do you feel that your health is significantly worse than last year?

① Obvious ② Less obvious ③ Not obvious

**1.2** Have you been eating a lot less recently?

① Obvious ② Less obvious ③ Not obvious

**1.3** Have you lost any sudden, noticeable weight recently? (“Significant weight loss” means a weight loss of at least 6kg in the last six months or at least 3 kg in the last one month)

① Obvious ② Less obvious ③ Not obvious

**1.4** Do you have difficulty moving your limbs because of the inconvenience in your daily life?

① Yes ② Occasionally ③ No

**1.5** Do you have difficulty maintaining your balance in your daily life?

① Yes ② Occasionally ③ No

**1.6** Do you have a language barrier that makes your daily life difficult?

① Yes ② Occasionally ③ No

**1.7** Do you have physical fatigue that makes your daily life difficult?

① Yes ② Occasionally ③ No

**1.8** Do you often feel tightness in your chest?

① Yes ② Occasionally ③ No

**1.9** Do you often feel physical pain? (pain in the limbs, head, neck, back, muscles, etc.)

① Yes ② Occasionally ③ No

**2. Mental Frailty**

**2.1** Have you been feeling down (unhappy) in the last month?

① Yes ② Occasionally ③ No

**2.2** Do you have physical fatigue that makes your daily life difficult?

① Yes ② Occasionally ③ No

**2.3** Have you been able to deal with the problems in your life in the last month?

① Able ② Can sometimes ③ Unable

**2.4** Have you been able to enlighten yourself on the problems you have encountered in the last month?

① Able ② Can sometimes ③ Unable

**2.5** Overall, are you satisfied with your current life or job?

① Satisfaction ② Partial satisfaction ③ Dissatisfaction

**3. Social Frailty**

**3.1** Do you live alone?

① Yes ② No

**3.2** Do you often invite people to your home? (including friends, relatives, neighbors, etc.)

① Yes ② Sometime ③ No

**3.3** Do you often visit other people's homes? (including friends, relatives, neighbors, etc.)

① Yes ② Sometime ③ No

**3.4** Do you think there are a lot of friends and relatives you can meet and confide in at any time? (including children, relatives, neighbors and friends)

① More ② Sometime ③ No

**3.5** Do you often feel lonely?

① Yes ② Sometime ③ No

**4. Environmental Frailty**

**4.1** Do you think your house is in bad condition?

① Bad ② Not too bad ③ Not bad

**4.2** Do you think your accommodation is not comfortable enough?

① Uncomfortable ② Not feel well ③ Comfortable

**4.3** Don't you like the surroundings of the house?

① Dislike ② Not too like ③ Like

**4.4** Do you think the transportation around your house is not easy?

① Yes ② Sometime ③ No

**Self-Perceived eHealth Literacy Status**

**D1 The application ability of ehealth information and service**

D1.1 I know how to find useful health information on the Internet.

①very inconsistent ②some inconsistent ③ambiguous ④some consistent ⑤very consistent

D1.2 I know how to solve health problems on the Internet.

①very inconsistent ②some inconsistent ③ambiguous ④some consistent ⑤very consistent

D1.3 I know what health information is available on the Internet.

①very inconsistent ②some inconsistent ③ambiguous ④some consistent ⑤very consistent

D1.4 I know where to get useful health information on the Internet.

①very inconsistent ②some inconsistent ③ambiguous ④some consistent ⑤very consistent

D1.5 I know how to make use of health information on the Internet.

①very inconsistent ②some inconsistent ③ambiguous ④some consistent ⑤very consistent

**D2 The judgment ability of ehealth information and service**

D2.1 I can evaluate the quality of health information on the Internet.

①very inconsistent ②some inconsistent ③ambiguous ④some consistent ⑤very consistent

D2.2 I can distinguish the quality of health information on the Internet.

①very inconsistent ②some inconsistent ③ambiguous ④some consistent ⑤very consistent

**D3 The determination ability of ehealth information and service**

D3.1 I am confident to make health-related decisions on the Internet.

①very inconsistent ②some inconsistent ③ambiguous ④some consistent ⑤very consistent

**Morisky Medication Adherence Scale**

**1. Do you ever forget to take your medicine?**

①Yes ②No

**2. Are you careless at times about taking your medicine?**

①Yes ②No

**3. When you feel better, do you sometimes stop taking your medicine?**

①Yes ②No

**4. Sometimes if you feel worse when you take the medicine, do you stop taking it?**

①Yes ②No
